# A new approach to keratoconus detection based on corneal morphogeometric analysis

**DOI:** 10.1371/journal.pone.0184569

**Published:** 2017-09-08

**Authors:** Francisco Cavas-Martínez, Laurent Bataille, Daniel G. Fernández-Pacheco, Francisco J. F. Cañavate, Jorge L. Alió

**Affiliations:** 1 Department of Graphical Expression, Technical University of Cartagena, Cartagena, Spain; 2 Research and Development Department, Vissum Corporation Alicante, Spain; 3 Keratoconus Unit, Vissum Corporation Alicante, Spain; 4 Division of Ophthalmology, Universidad Miguel Hernández, Alicante, Spain; 5 Department of Refractive Surgery, Vissum Corporation Alicante, Spain; Save Sight Institute, AUSTRALIA

## Abstract

**Purpose:**

To characterize corneal structural changes in keratoconus using a new morphogeometric approach and to evaluate its potential diagnostic ability.

**Methods:**

Comparative study including 464 eyes of 464 patients (age, 16 and 72 years) divided into two groups: control group (143 healthy eyes) and keratoconus group (321 keratoconus eyes). Topographic information (Sirius, CSO, Italy) was processed with SolidWorks v2012 and a solid model representing the geometry of each cornea was generated. The following parameters were defined: anterior (A_ant_) and posterior (A_post_) corneal surface areas, area of the cornea within the sagittal plane passing through the Z axis and the apex (A_apexant_, A_apexpost_) and minimum thickness points (A_mctant_, A_mctpost_) of the anterior and posterior corneal surfaces, and average distance from the Z axis to the apex (D_apexant_, D_apexpost_) and minimum thickness points (D_mctant_, D_mctpost_) of both corneal surfaces.

**Results:**

Significant differences among control and keratoconus group were found in A_apexant_, A_apexpost_, A_mctant_, A_mctpost_, D_apexant_, D_apexpost_ (all p<0.001), A_post_ (p = 0.014), and D_mctpost_ (p = 0.035). Significant correlations in keratoconus group were found between A_ant_ and A_post_ (r = 0.836), A_mctant_ and A_mctpost_ (r = 0.983), and D_mctant_ and D_mctpost_ (r = 0.954, all p<0.001). A logistic regression analysis revealed that the detection of keratoconus grade I (Amsler Krumeich) was related to A_post_, A_tot_, A_apexant_, A_mctant_, A_mctpost_, D_apexpost_, D_mctant_ and D_mctpost_ (Hosmer-Lemeshow: p>0.05, R^2^ Nagelkerke: 0.926). The overall percentage of cases correctly classified by the model was 97.30%.

**Conclusions:**

Our morphogeometric approach based on the analysis of the cornea as a solid is useful for the characterization and detection of keratoconus.

## Introduction

Keratoconus is an ectatic corneal disorder characterized by progressive corneal thinning and structural weakening and resulting in corneal protrusion, irregular astigmatism, and decreased vision [1]. Several diagnostic criteria have been defined using a great variety of techniques and technologies [2]. Besides classical keratoconus biomicroscopic signs [3], the conical protrusion and infero-superior asymmetry associated to keratoconus can be easily detected by means of corneal topography [3–5]. Problems arises when very incipient stages of keratoconus are intended to be detected (subclinical keratoconus). In such cases, a more comprehensive analysis of corneal geometry is necessary as well as the consideration of other complementary descriptors, such as corneal aberrations, pachymetry, asphericity, or the analysis of corneal biomechanical properties [6–14].

The correlation between the anterior and posterior corneal shape in keratoconus has been also investigated [14, 15] and its potential diagnostic value have been also even evaluated [4]. A comprehensive analysis of this relationship can be performed by means of geometric modeling enabling the characterization of the human cornea [16]. We previously validated the use of some new indices based on an innovative morphogeometric modeling of the corneal structure for the detection of keratoconus [16]. The current study is a continuation of this research by confirming the diagnostic ability of the morphogeometric indices developed but in a larger sample of patients as well as by creating a new predictive model of detection of incipient keratoconus based on the combination of such indices.

## Material and methods

### Patients

This was a comparative study including 464 eyes of 464 patients ranging in age between 16 and 72 years old. Only one eye from each patient was randomly selected for the study according to a random number sequence (dichotomic sequence, 0 and 1) that was created with specific software in order to avoid the interference in the analysis of the correlation that often exists between the two eyes of the same person. This study was conducted at Vissum Corporation in Alicante (Spain). Two groups of eyes were differentiated depending if the keratoconus disease was present or not: control group, including 143 healthy eyes, and keratoconus group, including 321 eyes with the diagnosis of keratoconus. The inclusion criterion for the control group was healthy eyes that did not meet the exclusion criteria and diagnosis according to the standard criteria for keratoconus diagnosis in the keratoconus group [2, 3], which is the presence of an asymmetric bowtie pattern in corneal topography, a value of 100 or higher of the KISA index, a central keratometry (K-value) with different cut-off values to keratoconus suspect (>47.2 D), a inferior-superior asymmetry (I-S value) with a cut-off value of 1.4 D difference between average inferior and superior corneal powers at 3 mm from the center of the cornea, as well as other topographic indices (SRAX, KSS, KPI, CLMI) and at least one keratoconus sign on slit-lamp examination, such as stromal thinning, conical protusion on the cornea at the apex, Fleischer ring, Vogt striae or anterior stromal scar. Exclusion criteria in both groups were previous ocular surgery and any other active ocular disease. Patients with forme fruste keratoconus (with topographic alterations compatible with keratoconus but without apparent clinical alterations of this pathology (normal visual acuity = 1.00)) and patients with normal eyes but with a keratoconic contralateral eye were not included in this study. The study was approved by the Vissum Corporation ethics committee and was then performed in accordance with the ethical standards laid down in the Declaration of Helsinki (Seventh revision, October 2013, Fortaleza, Brasil). A supplementary file (S1 Table) including the following patients’ data is provided: age, gender, contact lens wear, both eyes affected, studied eye, K2 and central thickness.

### Examination protocol

All patients underwent a complete eye examination including the following tests: anamnesis, measurement of uncorrected (UDVA) and corrected (CDVA) distance visual acuity, manifest refraction, slit-lamp biomicroscopy, and corneal analysis by the Sirius system (Costruzione Strumenti Oftalmici, Italy). Repeatability of the topographic measurements provided by the Sirius system in keratoconic eyes are demonstrated in previous studies [17]. All tests were performed by a single experienced examiner. A minimum of three corneal topographies were successively obtained for each cornea and the best one (the topography with the highest acquisition quality for the Scheimpflug image and keratoscopy) selected to provide data for this study. All corneal topography files were exported in.csv format. Likewise, all cases were classified according to the Amsler-Krumeich grading system [1].

### Geometric modeling

**Fig 1:**
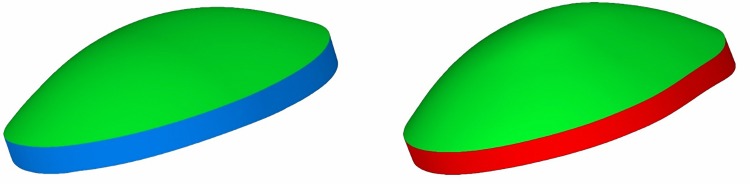
Area of the anterior corneal surface in the solid model generated for a specific cornea evaluated in the current study (green) compared to a healthy (blue) and keratoconus cornea (red).

**Fig 2:**
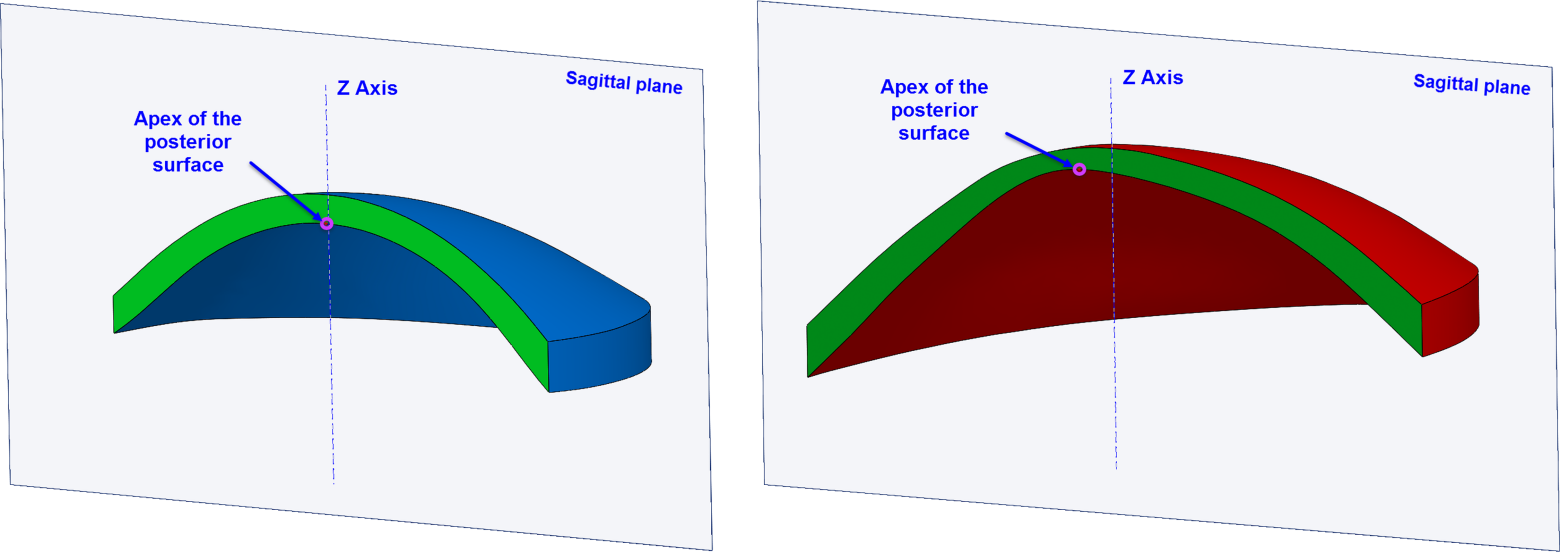
Area of the cornea within the sagittal plane passing through the Z axis and the highest point (apex) of the posterior corneal surface in a healthy (blue) and keratoconus cornea (red).

**Fig 3:**
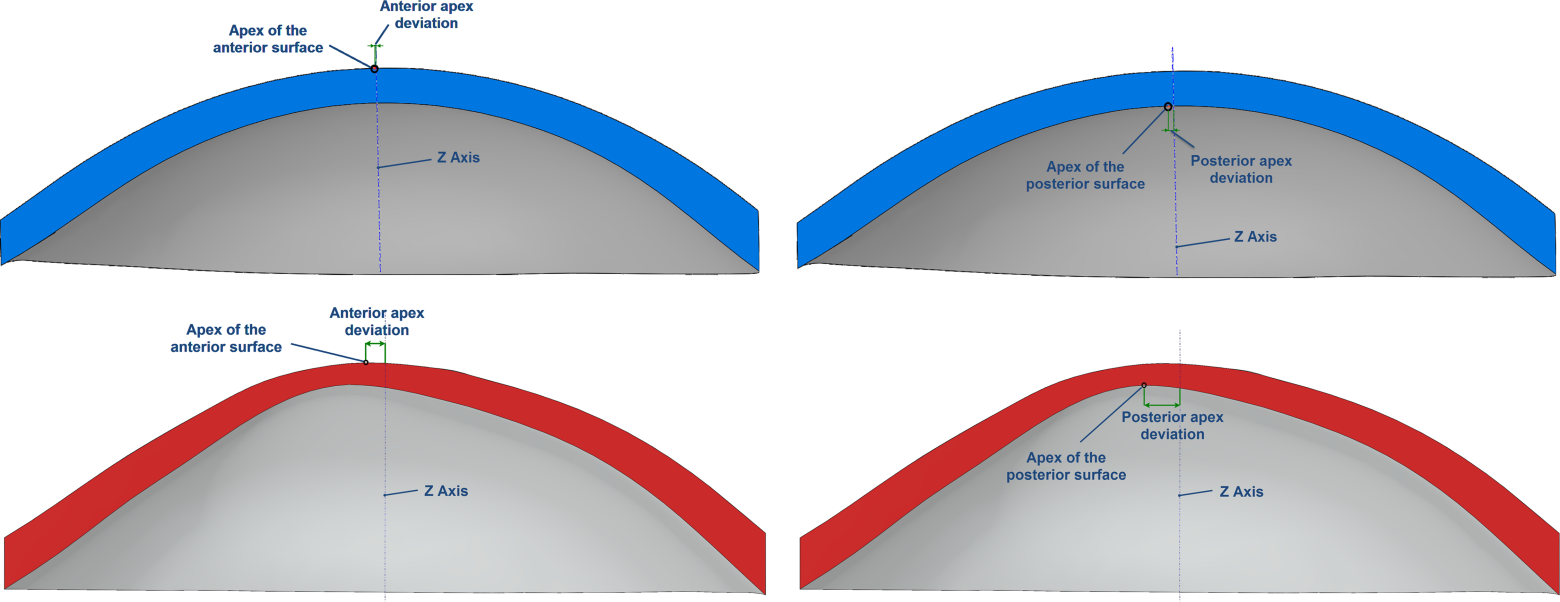
Average distance from the Z axis to the highest point (apex) of the anterior and posterior corneal surfaces in a healthy (blue) and keratoconus cornea (red).

**Fig 4:**
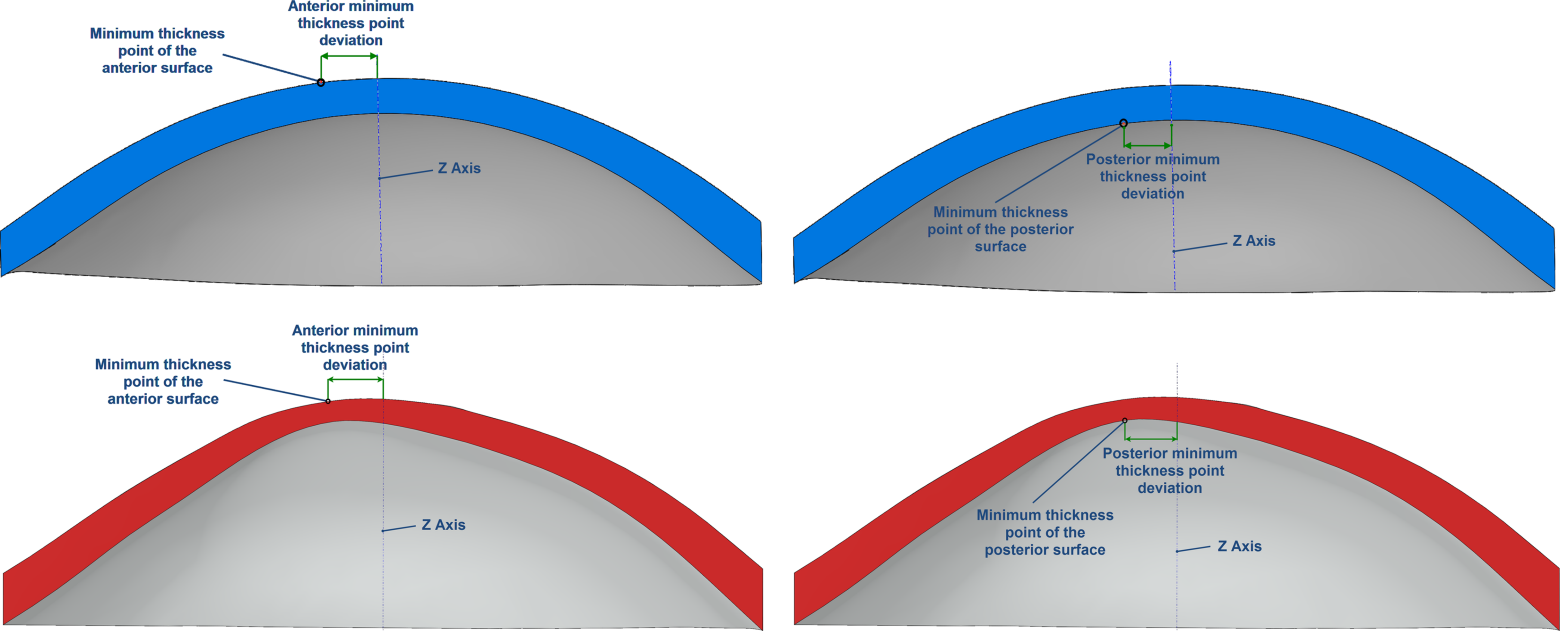
Average distance in the XY plane from the Z axis to the minimum thickness points (maximum curvature) of the anterior and posterior corneal surfaces in a healthy (blue) and keratoconus cornea (red).

The morphogeometric modeling was performed following a procedure previously described and validated by our research group [16]. In general terms, this method consisted of the following steps:

- Preparation of the point cloud. A surface from the geometry that a point cloud presents was generated in a coordinate system for a three-dimensional space. Topography files exported in.csv were formatted in Cartesian coordinates by an algorithm programmed using Matlab software. For such purpose, it was considered that every row represents a circle in the corneal map and every column represents a semi-meridian, providing a total of 256 points for each radius. Each i-th row sampled a map on a circle of i*0.2 mm radius, and each j-th column sampled a map on a semimeridian in the direction of j*360/256u, so each Z value of the matrix [i, j] represented the point P (i*0.2, j*360/256u) in polar coordinates. The geometric center of the cornea was obtained from the XYZ coordinates provided by the topographer, which correspond to the center of the Placido disc rings. Specifically, the point cloud was generated for the area from the corneal geometric center (r = 0 mm) to the beginning of the so-called peripheral zone (r = 4 mm). It should be taken into account that this area of analysis is considered to have more information on corneal morphology for both healthy and diseased eyes [16].
- Geometric Surface Reconstruction. The point cloud representing the corneal geometry was imported into the surface reconstruction software Rhinoceros v5.0. The surface that best fits the point cloud was generated with the Rhinoceros’s patch surface function that tries to minimize the nominal distance between the 3D point cloud and the solution surface. The settings of the function were configured as follows: sample point spacing 256, surface span planes 255 for both u and v directions, and stiffness of the solution surface.
- Solid Modeling. The resulting surface was imported into the solid modeling software SolidWorks v2012. With this software, the solid model representing the custom and actual geometry of each cornea was generated.
- Definition of the morphogeometric variables to analyze. From the solid model obtained, the following geometric variables were defined (Table 1):
  ○ Anterior corneal surface area (mm^2^) (A_ant_): area of the anterior corneal surface of the solid model generated (Fig 1)
  ○ Posterior corneal surface area (mm^2^) (A_post_): area of the posterior corneal surface of the solid model generated (Fig 1)
  ○ Total corneal surface area (mm^2^) (A_tot_): sum of anterior, posterior and perimetral corneal surface areas of the solid model generated
  ○ Sagittal plane apex area (mm^2^): area of the cornea within the sagittal plane passing through the Z axis and the highest point (apex) of the anterior (A_apexant_) or posterior (A_apexpost_) corneal surface (Fig 2)
  ○ Sagittal plane area at minimum thickness point (mm^2^): area of the cornea within the sagittal plane passing through the Z axis and the minimum thickness point of the anterior (A_mctant_) and posterior (A_mctpost_) corneal surfaces
  ○ Anterior and posterior apex deviation (mm): average distance from the Z axis to the highest point (apex) of the anterior (D_apexant_) and posterior corneal surfaces (D_apexpost_) (Fig 3)
  ○ Anterior and posterior minimum thickness point deviation (maximum curvature) (mm): average distance in the XY plane from the Z axis to the minimum thickness points (maximum curvature) of the anterior (D_mctant_) and posterior corneal surfaces (D_mctpost_) (Fig 4)

**Table 1:** List of acronyms used for the morphogeometric variables of the study.

Acronym	Description
**A_ant_**	Anterior corneal surface area
**A_post_**	Posterior corneal surface area
**A_tot_**	Total corneal surface area
**A_apexant_**	Sagittal plane area at anterior apex
**A_apexpost_**	Sagittal plane area at posterior apex
**A_mctant_**	Sagittal plane area at anterior minimum thickness point
**A_mctpost_**	Sagittal plane area at posterior minimum thickness point
**D_apexant_**	Anterior apex deviation
**D_apexpost_**	Posterior apex deviation
**D_mctant_**	Anterior minimum thickness point deviation
**D_mctpost_**	Posterior minimum thickness point deviation

### Statistical analysis

SPSS statistics software package version 15.0 (IBM, Armonk, EEUU) was used for the statistical analysis. Normality of all data was checked by means of the Kolmogorov-Smirnov test. A comparison between healthy and keratoconus groups was performed with the unpaired Student t or Mann-Whitney U tests depending if the data samples were normally distributed or not. An additional analysis was performed to compare differences between groups according to keratoconus stages graded using the Amsler-Krumeich classification system. The one-way analysis of variance (ANOVA) was used for such purpose if variables were normally distributed, whereas the Kruskal-Wallis test was used if one or more variables were not normally distributed. The post-hoc comparative analysis for the ANOVA was performed with the Bonferroni test when the variances were homogeneous and the T2 Tamhane test when the variances were not homogeneous, while the Mann-Whitney tests with the Bonferroni´s adjustment was used for the post-hoc analysis of the Kruskal-Wallis test. Pearson and Spearman correlation coefficients were used to assess the correlation between anterior and posterior geometric parameters depending if the data samples were or not normally distributed. Differences were considered to be statistically significant when the associated p-value was <0.05.

A stepwise backward logistic regression was also performed to define the key parameters involved in the detection of keratoconus grade I as moderate and severe keratoconus can be easily detected by means of topographic and biomicroscopic analysis. Hosmer-Lemeshow adjustment was used to assess the overall goodness of fit of the model, and R^2^ Cox and Snell and R^2^ Nagelkerke were used to study the variance rate explained by the variables of the model. The specific relationship between the parameters of the final model was evaluated with the model coefficients (B) and the odds ratios that represent the value of increased likelihood that a category of the dependent variable is met for each unit of the independent variable, while the other independent variables are held constant. Finally, the efficacy of the model to detect keratoconus grade I was compared with that provided by the classifier of the topography system used for obtaining the measurements. This classifier is based on the use of different indices obtained from both the anterior and posterior corneal surfaces, including symmetry index of front and back corneal curvature, best fit radius of the front corneal surface, Baiocchi Calossi Versaci front index (BCV(f)) and BCV back index (BCV(b)), root mean square of front and back corneal surface higher order aberrations, and thinnest corneal point [18].

## Results

A total of 143 healthy eyes of 143 patients (30.8%) (control group) and 321 keratoconus eyes of 321 patients (69.2%) (keratoconus group) were enrolled in the study. In the keratoconus group, the following subgroups were differentiated according to the stage of the disease following the Amsler-Krumeich grading system: grade I (229 eyes, 71.3%), grade II (59 eyes, 18.4%), grade III (9 eyes, 2.8%), and grade IV (24 eyes, 7.5%).

### Comparison control vs. keratoconus group

Table 2 summarizes the outcomes obtained in the control and keratoconus group. Significant differences among control and keratoconus group were found in A_post_ (p = 0.014), A_apexant_ (p<0.001), A_apexpost_ (p<0.001), A_mctant_ (p<0.001), A_mctpost_ (p<0.001), D_apexant_ (p<0.001), D_apexpost_ (p<0.001), and D_mctpost_ (p = 0.035). Specifically, in keratoconus group, significantly higher values of A_post_, A_apexant_, D_apexant_, D_apexpost_ and D_mctpost_ as well as lower values of A_apexpost_, A_mctant_ and A_mctpost_ compared to control group.

**Table 2:** Summary of the outcomes obtained in control and keratoconus groups.

Mean (SD) Median (Range)	Control	Keratoconus	p-valor (test)
A_ant_ (mm^2^)	43.08 (0.14) 43.08 (42.73 to 43.39)	43.12 (0.56) 43.00 (42.00 to 47.00)	0.435
A_post_ (mm^2^)	44.24 (0.28) 44.24 (43.49 to 44.90)	44.43 (0.89) 44.00 (43.00 to 51.00)	0.014
A_tot_ (mm^2^)	103.92 (1.20) 103.88 (100.69 to 106.15)	103.64 (1.91) 103.00 (99.96 to 114.00)	0.106
A_apexant_ (mm^2^)	0.24 (1.01) 0.00 (0.00 to 4.57)	1.99 (1.73) 3.00 (0.00 to 4.31)	<0.001
A_apexpost_ (mm^2^)	4.32 (0.26) 4.31 (3.58 to 5.00)	3.51 (0.52) 3.71 (2.00 to 5.00)	<0.001
A_mctant_ (mm^2^)	4.15 (0.37) 4.07 (3.00 to 5.01)	3.50 (0.52) 3.63 (2.00 to 5.00)	<0.001
A_mctpost_ (mm^2^)	4.31 (0.26) 4.32 (3.57 to 5.01)	3.50 (0.51) 3.65 (2.00 to 5.00)	<0.001
D_apexant_ (mm)	0.000 (0.001) 0.000 (0.000 to 0.007)	0.011 (0.017) 0.003 (0.000 to 0.070)	<0.001
D_apexpost_ (mm)	0.073 (0.053) 0.067 (0.024 to 0.650)	0.186 (0.095) 0.175 (0.011 to 0.594)	<0.001
D_mctant_ (mm)	0.879 (0.253) 0.844 (0.438 to 2.171)	0.907 (0.279) 0.973 (0.160 to 2.051)	0.314
D_mctpost_ (mm)	0.806 (0.235) 0.794 (0.375 to 2.059)	0.861 (0.266) 0.904 (0.104 to 2.000)	0.035

Abbreviations: SD, standard deviation; A_ant_, anterior corneal surface area; A_post_, posterior corneal surface area; A_tot_, total corneal surface area; A_apexant_ and A_apexpost_, area of the cornea within the sagittal plane passing through the Z axis and the highest point (apex) of the anterior or posterior corneal surface; A_mctant_ and A_mctpost_, area of the cornea within the sagittal plane passing through the Z axis and the minimum thickness point of the anterior and posterior corneal surfaces; D_apexant_ and D_apexpost_, average distance from the Z axis to the highest point (apex) of the anterior and posterior corneal surfaces; D_mctant_ and D_mctpost_, average distance in the XY plane from the Z axis to the minimum thickness points (maximum curvature) of the anterior and posterior corneal surfaces.

Table 3 summarizes the outcomes obtained in the control group and keratoconus subgroups according to the stage of severity of the disease. An extended version of the table is also provided as supplementary file (S2 Table). Significant differences among keratoconus stages were found in the geometric parameters evaluated (p<0.001). Specifically, significant differences were found among all keratoconus subgroups were found for A_post_ (p≤0.001). Significant differences were found between control group and keratoconus grade I subgroups for all parameters (p≤0.021) except for D_mctant_ (p≥0.056).

**Table 3:** Summary of the outcomes obtained in control group and keratoconus subgroups according to the stage of severity of the disease.

Mean (SD) Median (Range)	Control (C)	Ktc grade I (KC1)	Ktc grade II (KC2)	Ktc grade III (KC3)	Ktc grade IV (KC4)	p-valor (test)
A_ant_ (mm^2^)	43.08 (0.14) 43.08 (42.73 to 43.39)	42.93 (0.33) 43.00 (42.00 to 43.58)	43.29 (0.42) 43.00 (43.00 to 45.00)	43.97 (0.21) 44.00 (43.52 to 44.35)	44.22 (0.93) 44.00 (43.00 to 47.00)	<0.001
A_post_ (mm^2^)	44.24 (0.28) 44.24 (43.49 to 44.90)	44.07 (0.42) 44.00 (43.00 to 45.07)	44.84 (0.59) 45.00 (44.00 to 47.00)	45.60 (0.49) 45.78 (44.87 to 46.00)	46.37 (1.42) 46.00 (44.39 to 51.00)	<0.001
A_tot_ (mm^2^)	103.92 (1.20) 103.88 (100.69 to 106.15)	103.10 (1.33) 103.00 (100.00 to 107.00)	104.10 (1.43) 104.00 (99.96 to 109.00)	104.55 (1.97) 105.48 (101.00 to 106.00)	107.34 (2.93) 106.74 (103.00 to 114.00)	<0.001
A_apexant_ (mm^2^)	0.24 (1.01) 0.00 (0.00 to 4.57)	1.55 (1.78) 0.00 (0.00 to 4.31)	3.10 (0.98) 3.00 (0.00 to 4.00)	2.56 (1.01) 3.00 (0.00 to 3.00)	3.21 (0.83) 3.00 (0.00 to 4.00)	<0.001
A_apexpost_ (mm^2^)	4.32 (0.26) 4.31 (3.58 to 5.00)	3.55 (0.52) 3.95 (2.00 to 5.00)	3.47 (0.53) 3.56 (2.00 to 4.48)	3.04 (0.49) 3.00 (2.00 to 3.70)	3.37 (0.50) 3.00 (3.00 to 4.27)	<0.001
A_mctant_ (mm^2^)	4.15 (0.37) 4.07 (3.00 to 5.01)	3.54 (0.52) 3.87 (2.00 to 5.00)	3.46 (0.53) 3.53 (2.00 to 4.48)	3.04 (0.49) 3.00 (2.00 to 3.69)	3.33 (0.48) 3.00 (3.00 to 4.27)	<0.001
A_mctpost_ (mm^2^)	4.31 (0.26) 4.32 (3.57 to 5.01)	3.54 (0.52) 3.87 (2.00 to 5.00)	3.50 (0.49) 3.63 (3.00 to 4.48)	3.15 (0.30) 3.00 (2.99 to 3.69)	3.33 (0.48) 3.00 (3.00 to 4.27)	<0.001
D_apexant_ (mm)	0.000 (0.001) 0.000 (0.000 to 0.007)	0.006 (0.012) 0.000 (0.000 to 0.070)	0.022 (0.021) 0.012 (0.000 to 0.069)	0.022 (0.023) 0.014 (0.000 to 0.066)	0.024 (0.019) 0.019 (0.000 to 0.066)	<0.001
D_apexpost_ (mm)	0.073 (0.053) 0.067 (0.024 to 0.650)	0.170 (0.088) 0.160 (0.011 to 0.594)	0.211 (0.101) 0.197 (0.026 to 0.453)	0.217 (0.096) 0.221 (0.054 to 0.368)	0.266 (0.097) 0.290 (0.052 to 0.412)	<0.001
D_mctant_ (mm)	0.879 (0.253) 0.844 (0.438 to 2.171)	0.934 (0.266) 1.000 (0.336 to 2.051)	0.893 (0.317) 0.856 (0.307 to 1.828)	0.680 (0.250) 0.697 (0.233 to 1.000)	0.766 (0.247) 0.856 (0.160 to 1.000)	0.003
D_mctpost_ (mm)	0.806 (0.235) 0.794 (0.375 to 2.059)	0.889 (0.249) 0.953 (0.319 to 2.000)	0.840 (0.306) 0.791 (0.267 to 1.725)	0.633 (0.256) 0.631 (0.197 to 1.000)	0.728 (0.252) 0.809 (0.104 to 1.000)	<0.001

Abbreviations: SD, standard deviation; A_ant_, anterior corneal surface area; A_post_, posterior corneal surface area; A_tot_, total corneal surface area; A_apexant_ and A_apexpost_, area of the cornea within the sagittal plane passing through the Z axis and the highest point (apex) of the anterior or posterior corneal surface; A_mctant_ and A_mctpost_, area of the cornea within the sagittal plane passing through the Z axis and the minimum thickness point of the anterior and posterior corneal surfaces; D_apexant_ and D_apexpost_, average distance from the Z axis to the highest point (apex) of the anterior and posterior corneal surfaces; D_mctant_ and D_mctpost_, average distance in the XY plane from the Z axis to the minimum thickness points (maximum curvature) of the anterior and posterior corneal surfaces

### Correlation between anterior and posterior corneal geometry parameters

**Fig 5:**
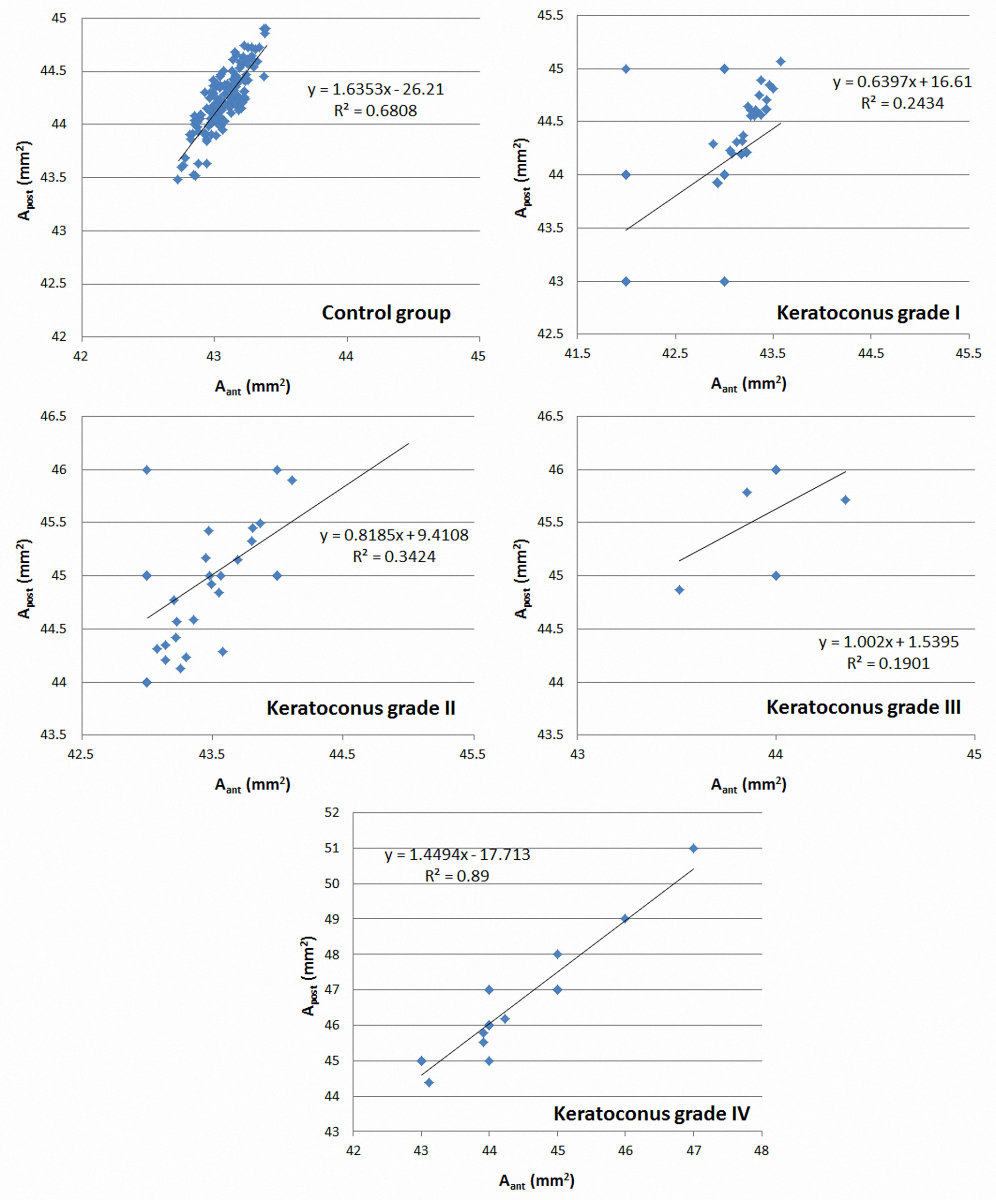
Scatterplots showing the relationship between the areas of the anterior corneal surface (A_ant_) and posterior corneal surface area (A_post_). The adjusting line to the data obtained by means of the least-squares fit is shown.

Table 4 summarizes the correlations obtained between the geometric parameters of the anterior and posterior corneal surfaces in the control group and keratoconus group as well as in the keratoconus subgroups according to the stage of severity of the disease. As shown, strong correlations among A_ant_ and A_post_ were found in both control and keratoconus groups. However, when the results are analyzed according to keratoconus severity, a stronger correlation between A_ant_ and A_post_ was observed in eyes with severe keratoconus compared to the rest (Fig 5). No significant correlations were found between A_apexant_ and A_apexpost_ in control group and keratoconus grade I, II and III subgroups. However, the correlation between these two parameters was strong in keratoconus grade IV subgroup. Similarly, the correlation between D_apexant_ and D_apexpost_ became stronger and statistically significant in keratoconus grade IV compared to the rest. Regarding the correlation between A_mctant_ and A_mctpost_, it was good and statistically significant in all keratoconus subgroups, but somewhat weaker in control group. A very strong correlation among D_mctant_ and D_mctpost_ was found in all groups and subgroups.

**Table 4:** Summary of the correlations obtained between the geometric parameters of the anterior and posterior corneal surfaces in the control group and keratoconus subgroups according to the stage of severity of the disease.

Correlation coefficient (p-value)	Control (C)	Keratoconus (KC)	Ktc grade I (KC1)	Ktc grade II (KC2)	Ktc grade III (KC3)	Ktc grade IV (KC4)
A_ant_-A_post_	0.825 (p<0.001)	0.836 (p<0.001)	0.493 (p<0.001)	0.585 (p<0.001)	0.436 (p = 0.241)	0.943 (p<0.001)
A_apexant_-A_apexpost_	0.051 (p = 0.544)	0.076 (p = 0.176)	0.125 (p = 0.058)	0.153 (p = 0.247)	-0.217 (p = 0.576)	0.567 (p = 0.004)
A_mctant_-A_mctpost_	0.677 (p<0.001)	0.983 (p<0.001)	0.999 (p<0.001)	0.938 (p<0.001)	0.742 (p = 0.022)	0.999 (p<0.001)
D_apexant_—D_apexpost_	0.031 (p = 0.712)	0.396 (p<0.001)	0.328 (p<0.001)	0.320 (p = 0.014)	-0.073 (p = 0.853)	0.478 (p = 0.018)
D_mctant_—D_mctpost_	0.982 (p<0.001)	0.954 (p<0.001)	0.931 (p<0.001)	0.994 (p<0.001)	0.993 (p<0.001)	0.987 (p<0.001)

Abbreviations: A_ant_, anterior corneal surface area; A_post_, posterior corneal surface area; A_apexant_ and A_apexpost_, area of the cornea within the sagittal plane passing through the Z axis and the highest point (apex) of the anterior or posterior corneal surface; A_mctant_ and A_mctpost_, area of the cornea within the sagittal plane passing through the Z axis and the minimum thickness point of the anterior and posterior corneal surfaces; D_apexant_ and D_apexpost_, average distance from the Z axis to the highest point (apex) of the anterior and posterior corneal surfaces; D_mctant_ and D_mctpost_, average distance in the XY plane from the Z axis to the minimum thickness points (maximum curvature) of the anterior and posterior corneal surfaces

### Predictive model for subclinical keratoconus detection

The logistic regression analysis revealed that the detection of keratoconus grade I was related to the variables A_post_, A_tot_, A_apexant_, A_mctant_, A_mctpost_, D_apexpost_, D_mctant_ and D_mctpost_ (p>0.05, Chi-Square and Hosmer-Lemeshow). The coefficient of determination R^2^ Cox and Snell (general) was 0.681, while the R^2^ Nagelkerke (corrected) was 0.926. Table 5 shows the model coefficients (B), the statistical significance, the exponential of B (ExpB, odds ratio) and confidence interval 95% of ExpB for each variable in the model. Specifically, the model revealed that the probability of having keratoconus grade I is 79.91 times higher for each mm^2^ increase of A_tot_, 1.77 times higher for each mm^2^ increase of A_apexant_, 5.99 x 10^31^ times higher for each mm^2^ increase of A_mctant_, 1.58 x 10^9^ times higher for each mm increase of D_apexpost_, and 5.511 x 10^7^ times higher for each mm increase of D_mctpost_. The overall percentage of cases correctly classified by our model was 97.30% (97.2% control group, 97.4% keratoconus grade I subgroup), whereas the percentage of cases correctly identified by the classifier of the topography system was 91.94% (97.9% control group, 88.2%).

**Table 5:** Summary of model defined for detection of early keratoconus.

	B	Sig	ExpB	CI 95% for ExpB
**A_post_ (mm^2^)**	-8.99	0.001	1.250x10^-4^	6.200x10^-7^ to 0.025
**A_tot_ (mm^2^)**	4.38	<0.001	79.911	8.518 to 749.706
**A_apexant_ (mm^2^)**	0.57	0.009	1.768	1.155 to 2.706
**A_mctant_ (mm^2^)**	73.17	0.256	5.989x10^31^	9.086x10^-24^ to 3.947x10^86^
**A_mctpost_ (mm^2^)**	-96.94	0.135	7.947x10^-43^	4.420x10^-98^ to 1.429x10^13^
**D_apexpost_ (mm)**	21.18	0.001	1.576x10^9^	7.153x10^3^ to 3.473x10^14^
**D_mctant_ (mm)**	-17.33	0.052	2.982x10^-8^	7.859x10^-16^ to 1.132
**D_mctpost_ (mm)**	17.83	0.058	5.511x10^7^	0.532 to 5.705x10^15^
**Constant of the model**	40.06	0.358	2.486x10^17^	

Abbreviations: A_post_, posterior corneal surface area; A_tot_, total corneal surface area; A_apexant_, area of the cornea within the sagittal plane passing through the Z axis and the highest point (apex) of the anterior corneal surface; A_mctant_ and A_mctpost_, area of the cornea within the sagittal plane passing through the Z axis and the minimum thickness point of the anterior and posterior corneal surfaces; D_apexpost_, average distance from the Z axis to the highest point (apex) of the posterior corneal surface; D_mctant_ and D_mctpost_, average distance in the XY plane from the Z axis to the minimum thickness points (maximum curvature) of the anterior and posterior corneal surfaces

## Discussion

The development of more sensitive algorithms for the detection of most incipient cases of keratoconus is currently of great interest as there are several therapeutic options that would allow halting the progression of the disease. In this line, we have tried to define a new predictive model for the detection of incipient keratoconus but based on a previously developed morphogeometric modeling of the cornea [16]. There is already a great variety of indices and diagnostic systems defined for the detection of keratoconus [2], but most of them are based on the analysis of curvature changes or asymmetries of both posterior corneal surfaces [4, 5, 8, 14, 15]. Likewise, corneal elevation [6, 7], corneal aberrometric [11], and pachymetric algorithms [10] have been also defined for the detection of keratoconus as well as the indirect measurement of some biomechanical parameters [9, 12]. However, few approaches have been developed for keratoconus detection considering the cornea as a solid with a specific volume [14], including an evaluation of the relationship between different sections of this solid [16]. This type of analysis may contribute to a better differentiation between healthy and pathological corneas and supposes a new concept in the characterization of the corneal structure in keratoconus. In our approach, we have defined and analyzed the following variables: the areas of the anterior and posterior corneal surfaces of the solid model generated (A_ant_, A_post_), the total corneal surface area (A_tot_), the areas of the cornea within the sagittal plane passing through the Z axis and the highest point of both corneal surfaces (A_apexant_, A_apexpost_), the areas of the cornea within the sagittal plane passing through the Z axis and the minimum thickness point of both corneal surfaces (A_mctant_, A_mctpost_) corneal surfaces, the average distance from the Z axis to the highest point of the anterior and posterior corneal surfaces (D_apexant_, D_apexpost_), and the average distance in the XY plane from the Z axis to the minimum thickness points of the anterior and posterior corneal surfaces (D_mctant_, D_mctpost_). We demonstrated in a previous study evaluating a significantly smaller sample of eyes that some of these parameters provided a good diagnostic ability for the detection of keratoconus (A_ant_, A_post_, A_apexant_, and A_apexpost_) [16]. The current study was aimed at studying the distribution of the new geometric variables defined in the healthy and keratoconus population and to define a new model of detection of early keratoconus based on the combination of these variables.

In our sample of 143 healthy eyes and 321 eyes with keratoconus, we found significant differences among groups in A_post_, A_apexant_, A_apexpost_, A_mctant_, A_mctpost_, D_apexant_, D_apexpost_, and D_mctpost_. This is consistent with the results found in our previous preliminary study conducted on 41 keratoconus eyes [16]. These results confirms that changes occurring in posterior corneal surface in keratoconus lead to an increase of the area corresponding to such surface (A_post_). This is mainly due to the localized steepening that occur in this surface in the area of the cone, with no significant flattening in the periphery [4, 5, 14, 15]. This generates that the area occupied by this surface would be significantly higher. In contrast, this does not happen with A_ant_, as steepening areas in anterior corneal surface in keratoconus are not so relevant in incipient stages but are very marked in moderate and advanced cases, which introduces a significant variability in the analysis of this parameter [2, 4, 5, 14, 15].

The analysis of A_apexant_ data revealed that a significantly lower value of this area was present in healthy eyes compared to keratoconus. However, the trend was the opposite for A_apexpost_, with lower values in keratoconus group. This is consistent with changes in the position of the apex of the anterior corneal surface in keratoconus eyes. It should be considered that the point of maximum elevation in keratoconus is displaced in most of cases inferiorly close to or coincident with the point of minimum thickness, even in incipient cases [2, 19–21]. Abu Ameerh et al [20] found in a sample of 210 patients with keratoconus that the vertical apex location correlated well with severity levels while the horizontal location seemed to have no effect. In our sample, A_apexant_ increased as the severity of keratoconus was higher, confirming that the displacement of the apex of the anterior corneal surface is an effect related to the progression of the disease. However, this trend was not observed for A_apexpost_, with higher values in the control group compared to the keratoconus group and subgroups. This suggests that there is some level of displacement of the posterior corneal apex compared to the sagittal plane passing through the Z axis in the posterior corneal surface in the healthy eye. Therefore, a change in this displacement would a sign related to the corneal ectatic process. This is consistent with studies reporting an asymmetric distribution of corneal pachymetry derived from the relationship among the elevation of both corneal surfaces in normal healthy corneas [22]. Jonuscheit et al [22] demonstrated that the nasal-temporal asymmetry of 113 healthy eyes became greater with increasing distance from the corneal center, with a mean difference of 59 ± 22 μm at 4 mm from the apex.

Regarding A_mctant_ and A_mctpost_, we found that both areas were significantly lower in keratoconus corneas compared to control group, with no clear differences among keratoconus severity subgroups. This suggests that although the position of minimal thickness is altered in keratoconus, this alteration is not coincident with that observed in the corneal apex or the point of maximum elevation. Indeed, Auffarth et al [21] demonstrated several years ago that there was a significant distance in keratoconus corneas between the apex and the thinnest point. Specifically, they found a mean value for this distance of 0.917 ± 0.729 mm [21]. Therefore, we cannot state that the point of minimum thickness and the corneal apex are coincident in all keratoconus cases. Indeed, significant differences among keratoconus and control groups were found in our sample for D_apexant_ and D_apexpost_, which is the distance from the Z axis to the point of maximum height (apex) of the anterior and posterior corneal surfaces, but not for D_mctant_, which is the distance from the Z axis to the point of minimum corneal thickness of the anterior corneal surface.

Significant correlations were found among the parameters defined for anterior and posterior corneal surfaces, except for the correlations between A_apexant_ and A_apexpost_, and also between D_apexant_ and D_apexpost_. This confirms that changes occurring in the points corresponding to the minimum corneal thickness in the anterior and posterior corneal surfaces as well as the area of each surface are correlated. This is consistent with the results of previous studies evaluating the correlation of standard geometric parameters of anterior and posterior corneal surfaces in keratoconus [4, 5, 14, 15]. Our research group have found in previous studies correlations between anterior and posterior corneal surfaces in terms of curvature, asphericity and astigmatism [4, 14, 15]. Curvature and asphericity changes of both corneal surfaces in keratoconus are related to the area of each surface and for this reason we also obtained a correlation between the areas calculated with our geometric approach for both surfaces. Furthermore, we found in our series an increased strength of the correlation between A_ant_ and A_post_ as the severity of the keratoconus increases, suggesting that an increasing area of steepening surrounded by an increasing area of corneal flattening is present as the severity of the disease is also increased. This is consistent with the definition of the course of the disease [1, 2] and therefore confirms that our approach is also reflecting the changes occurring in keratoconus but from another perspective. As a new finding, we have confirmed that changes in the apex of both corneal surfaces are not correlated in keratoconus, except for those eyes with an advanced stage of the disease. To our knowledge, this is the first study reporting this outcome.

Finally, we have obtained by logistic regression a new model of detection of early keratoconus (only grade I) considering the new parameters defined according to our new geometric approach. It should be considered that moderate and advanced keratoconus can be easily detected by means of conventional topographic analyses and the real challenge is to detect with accuracy those cases of keratoconus in an incipient stage. We found that considering A_post_, A_tot_, A_apexant_, A_mctant_, A_mctpost_, D_apexpost_, D_mctant_ and D_mctpost_, a detection of keratoconus grade I could be done with sensitivity of 97.4% and specificity of 97.2%. However, the percentage of cases correctly identified by the classifier of the topography system was 91.94%. The levels of sensitivity and specificity found are equivalent and even better than those reported by other different models of keratoconus detection based on standard topographic analysis [18, 19, 23–27]. The cone location and magnitude index (CLMI) combining different topographic parameters has shown to provide a keratoconus detection accuracy of 92% [23]. The SCORE Analyzer which integrates the data obtained with a scanning-slit topography system allows the detection of keratoconus with sensitivity and specificity of 92% and 96%, respectively [27]. The Sirius topography system includes a keratoconus detection analysis that has been reported to provide true predictions in around 93% of cases or more [18]. In our sample, the percentage of true predictions was close to 92%, a value below the true predictions obtained with our model. Future studies should confirm if our model of detection can be also applied with accuracy for the detection of subclinical keratoconus or if some adjustments are necessary to optimize the levels of sensitivity and specificity of the model.

In conclusion, a new geometric approach based on the analysis of the cornea as a solid with a specific volume, including an evaluation of the relationship between different sections of this solid, is a useful tool for the detection of keratoconus. The use of the combination of a variety of parameters based on this geometric approach is highly sensitive and specific for an accurate detection of incipient keratoconus cases.

## Supporting information

S1 Table Demographics of the study population (control and keratoconus). (XLSX)

S2 Table Summary of the outcomes obtained in control group and keratoconus subgroups according to the stage of severity of the disease (extended information). (DOCX)
