# Supplementary material for: A new approach to keratoconus detection based on corneal morphogeometric analysis
Source: PLoS One. 2017 Sep 8;12(9):e0184569. doi: 10.1371/journal.pone.0184569 (PMC5590974; doi:10.1371/journal.pone.0184569)
Supplement: S2 Table — (DOCX) [file pone.0184569.s002.docx]

**S2 Table**. Summary of the outcomes obtained in control group and keratoconus subgroups according to the stage of severity of the disease (extended information).

| Mean (SD)  Median (Range) | Control (C) | Ktc grade I (KC1) | Ktc grade II (KC2) | Ktc grade III (KC3) | Ktc grade IV (KC4) | p-valor  (test) |  |
| --- | --- | --- | --- | --- | --- | --- | --- |
| A_ant_ (mm^2^) | 43.08 (0.14)  43.08 (42.73 to 43.39) | 42.93 (0.33)  43.00 (42.00 to 43.58) | 43.29 (0.42)  43.00 (43.00 to 45.00) | 43.97 (0.21)  44.00 (43.52 to 44.35) | 44.22 (0.93)  44.00 (43.00 to 47.00) | <0.001 | C-KC1 0.001 C-KC2 0.002 KC3-4 0.760 Rest <0.001 |
| A_post_ (mm^2^) | 44.24 (0.28)  44.24 (43.49 to 44.90) | 44.07 (0.42)  44.00 (43.00 to 45.07) | 44.84 (0.59)  45.00 (44.00 to 47.00) | 45.60 (0.49)  45.78 (44.87 to 46.00) | 46.37 (1.42)  46.00 (44.39 to 51.00) | <0.001 | C-KC1 0.021 KC3-4 0.001 Rest <0.001 |
| A_tot_ (mm^2^) | 103.92 (1.20)  103.88 (100.69 to 106.15) | 103.10 (1.33)  103.00 (100.00 to 107.00) | 104.10 (1.43)  104.00 (99.96 to 109.00) | 104.55 (1.97)  105.48 (101.00 to 106.00) | 107.34 (2.93)  106.74 (103.00 to 114.00) | <0.001 | C-KC2 0.999 C-KC3 0.999 KC1-3 0.032 KC2-3 0.999 Rest <0.001 |
| A_apexant_ (mm^2^) | 0.24 (1.01)  0.00 (0.00 to 4.57) | 1.55 (1.78)  0.00 (0.00 to 4.31) | 3.10 (0.98)  3.00 (0.00 to 4.00) | 2.56 (1.01)  3.00 (0.00 to 3.00) | 3.21 (0.83)  3.00 (0.00 to 4.00) | <0.001 | KC1-3 0.402 KC2-3 0.999 KC2-4 0.999 KC3-4 0.999 Rest <0.001 |
| A_apexpost_ (mm^2^) | 4.32 (0.26)  4.31 (3.58 to 5.00) | 3.55 (0.52)  3.95 (2.00 to 5.00) | 3.47 (0.53)  3.56 (2.00 to 4.48) | 3.04 (0.49)  3.00 (2.00 to 3.70) | 3.37 (0.50)  3.00 (3.00 to 4.27) | <0.001 | KC1-2 0.999 KC1-3 0.010 KC1-4 0.628 KC2-3 0.089 KC3-4 0.633 KC2-4 0.999 Rest <0.001 |
| A_mctant_ (mm^2^) | 4.15 (0.37)  4.07 (3.00 to 5.01) | 3.54 (0.52)  3.87 (2.00 to 5.00) | 3.46 (0.53)  3.53 (2.00 to 4.48) | 3.04 (0.49)  3.00 (2.00 to 3.69) | 3.33 (0.48)  3.00 (3.00 to 4.27) | <0.001 | KC1-2 0.999 KC1-3 0.021 KC1-4 0.417 KC2-3 0.131 KC2-4 0.999 KC3-4 0.999 Rest <0.001 |
| A_mctpost_ (mm^2^) | 4.31 (0.26)  4.32 (3.57 to 5.01) | 3.54 (0.52)  3.87 (2.00 to 5.00) | 3.50 (0.49)  3.63 (3.00 to 4.48) | 3.15 (0.30)  3.00 (2.99 to 3.69) | 3.33 (0.48)  3.00 (3.00 to 4.27) | <0.001 | KC1-2 0.999 KC1-3 0.104 KC1-4 0.296 KC2-3 0.301 KC2-4 0.999 KC3-4 0.999 Rest <0.001 |
| D_apexant_ (mm) | 0.000 (0.001)  0.000 (0.000 to 0.007) | 0.006 (0.012)  0.000 (0.000 to 0.070) | 0.022 (0.021)  0.012 (0.000 to 0.069) | 0.022 (0.023)  0.014 (0.000 to 0.066) | 0.024 (0.019)  0.019 (0.000 to 0.066) | <0.001 | KC1-3 0.001 KC2-3 0.999 KC3-4 0.999 KC2-4 0.999 Rest <0.001 |
| D_apexpost_ (mm) | 0.073 (0.053)  0.067 (0.024 to 0.650) | 0.170 (0.088)  0.160 (0.011 to 0.594) | 0.211 (0.101)  0.197 (0.026 to 0.453) | 0.217 (0.096)  0.221 (0.054 to 0.368) | 0.266 (0.097)  0.290 (0.052 to 0.412) | <0.001 | KC1-2 0.005 KC1-3 0.866 KC2-3 0.999 KC2-4 0.056 KC3-4 0.999 Rest <0.001 |
| D_mctant_ (mm) | 0.879 (0.253)  0.844 (0.438 to 2.171) | 0.934 (0.266)  1.000 (0.336 to 2.051) | 0.893 (0.317)  0.856 (0.307 to 1.828) | 0.680 (0.250)  0.697 (0.233 to 1.000) | 0.766 (0.247)  0.856 (0.160 to 1.000) | 0.003 | Only significant KC1-4 0.038 Rest ≥0.056 |
| D_mctpost_ (mm) | 0.806 (0.235)  0.794 (0.375 to 2.059) | 0.889 (0.249)  0.953 (0.319 to 2.000) | 0.840 (0.306)  0.791 (0.267 to 1.725) | 0.633 (0.256)  0.631 (0.197 to 1.000) | 0.728 (0.252)  0.809 (0.104 to 1.000) | <0.001 | Only significant C-KC1 0.023 KC1-3 0.031 KC1-4 0.032 Rest ≥0.228 |

Abbreviations: SD, standard deviation; A_ant_, anterior corneal surface area; A_post_, posterior corneal surface area; A_tot_, total corneal surface area; A_apexant_ and A_apexpost_, area of the cornea within the sagittal plane passing through the Z axis and the highest point (apex) of the anterior or posterior corneal surface; A_mctant_ and A_mctpost_, area of the cornea within the sagittal plane passing through the Z axis and the minimum thickness point of the anterior and posterior corneal surfaces; D_apexant_ and D_apexpost_, average distance from the Z axis to the highest point (apex) of the anterior and posterior corneal surfaces; D_mctant_ and D_mctpost_, average distance in the XY plane from the Z axis to the minimum thickness points (maximum curvature) of the anterior and posterior corneal surfaces
